# Molecular perturbations in pulmonary tuberculosis patients identified by pathway-level analysis of plasma metabolic features

**DOI:** 10.1371/journal.pone.0262545

**Published:** 2022-01-24

**Authors:** Nguyen Phuoc Long, Da Young Heo, Seongoh Park, Nguyen Thi Hai Yen, Yong-Soon Cho, Jae-Gook Shin, Jee Youn Oh, Dong-Hyun Kim

**Affiliations:** 1 Center for Personalized Precision Medicine of Tuberculosis, Inje University College of Medicine, Busan, Republic of Korea; 2 Department of Pharmacology and PharmacoGenomics Research Center, Inje University College of Medicine, Busan, Republic of Korea; 3 Department of Statistics, Sungshin Women’s University, Seoul, Republic of Korea; 4 Department of Clinical Pharmacology, Inje University Busan Paik Hospital, Busan, Republic of Korea; 5 Division of Pulmonary, Allergy and Critical Care Medicine, Department of Internal Medicine, Korea University Guro Hospital, Seoul, Republic of Korea; Rutgers Biomedical and Health Sciences, UNITED STATES

## Abstract

Insight into the metabolic biosignature of tuberculosis (TB) may inform clinical care, reduce adverse effects, and facilitate metabolism-informed therapeutic development. However, studies often yield inconsistent findings regarding the metabolic profiles of TB. Herein, we conducted an untargeted metabolomics study using plasma from 63 Korean TB patients and 50 controls. Metabolic features were integrated with the data of another cohort from China (35 TB patients and 35 controls) for a global functional meta-analysis. Specifically, all features were matched to a known biological network to identify potential endogenous metabolites. Next, a pathway-level gene set enrichment analysis-based analysis was conducted for each study and the resulting *p*-values from the pathways of two studies were combined. The meta-analysis revealed both known metabolic alterations and novel processes. For instance, retinol metabolism and cholecalciferol metabolism, which are associated with TB risk and outcome, were altered in plasma from TB patients; proinflammatory lipid mediators were significantly enriched. Furthermore, metabolic processes linked to the innate immune responses and possible interactions between the host and the bacillus showed altered signals. In conclusion, our proof-of-concept study indicated that a pathway-level meta-analysis directly from metabolic features enables accurate interpretation of TB molecular profiles.

## Introduction

Tuberculosis (TB) is a devastating infectious disease, and an estimated 1.7 billion people are latently infected globally [1]. Despite extensive efforts, TB remains a leading cause of mortality worldwide, especially in countries where it is endemic. According to the World Health Organization Global Report (2020), there were around 10 million newly diagnosed TB patients in 2019, and approximately 1.4 million deaths [2]. TB has a broad pathophysiological spectrum, hampering eradication efforts [3]. A holistic model based on high-dimensional data is required to describe host-response endotype characteristics in general, and the TB immune endotype in particular. Specifically, -omics technologies have facilitated the discovery of clinically useful biomarkers for risk assessment, diagnosis, and prediction of clinical events. For instance, after performing a comprehensive analysis of plasma pulmonary TB samples, and samples from community-acquired pneumonia patients, lung cancer patients and normal controls, Huang *et al*. introduced xanthine, 4-pyridoxate, and d-glutamic acid as potential biomarkers [4]. Sweeney3 (*GBP5*, *DUSP3*, *KLF2*), a host-response-based gene signature, met the criteria of the World Health Organization/Foundation for Innovative New Diagnostics target product profile for a non-sputum-based triage test [5]. Comprehensive -omics data and appropriate analytical methods enable investigation of drug efficacy, personalized dosing, prediction of relapse-free cure, and phenotypic drug susceptibility testing, as aspects of personalized precision medicine [6–8].

Studies of host-response transcriptome biosignatures have achieved considerable success in terms of stratifying TB patients for the purposes of risk prediction [9], diagnosis [10], treatment monitoring, outcome prediction [11], and recurrence prediction [12]. Blood metabolic responses have also been tracked based on the “blood metabolic signature,” which partially reflects the interaction between the human body and *Mycobacterium tuberculosis* (*M*. *tuberculosis*) bacilli. The metabolic responses of TB patients may aid predictions of risk, diagnosis, and outcomes, as well as treatment monitoring [13]. Integrating multi-omics data with clinical information could facilitate host-directed therapy for TB; for example, TB meningitis [14]. However, the usefulness of the serum and plasma metabolomic analysis for tracking the blood metabolic signature of TB across populations is unclear. Moreover, the variability in study designs and limited guidelines for the use of omics technologies in clinical research could lead to less reliable data, complicated analyses, and missed biological signals [15]. Therefore, rigorous designs are required for the reproducibility of–omics studies.

In computationally functional interpretation, a set of genes or metabolites associated with a phenotype of interest is typically identified by a statistical test. Next, it is compared with a predefined database of biological functions, which returns enriched scores, for which *p*-values and/or q-values are calculated. Its fundamental principle comprises over-representation analyses. Gene set enrichment analysis (GSEA) utilizes a metric representing the overall ranks of features (e.g., t-score or fold-change) to find “significantly coordinated reposition” of the association strength based on a database of genes or metabolites sharing biological functions [16]. GSEA has been used extensively in transcriptomics studies, but comparatively infrequently in metabolomics. Despite its ability to obtain profound information from samples, untargeted metabolomics has not met expectations in terms of providing mechanistic insight into the metabolic alterations of phenotypes of interest. This is primarily due to the difficulty of compound annotation and identification. Generally, a tiny fraction of ions can be assigned to metabolites with an acceptable level of confidence. The limited ability to define metabolites hampers subsequent functional interpretation. The situation has gradually improved since the introduction of the Mummichog algorithm [17]. Overall, Mummichog leverages known metabolic networks to map all potentially molecular relevant metabolites. Hence, it allows a test of representation in which potentially valid metabolites are over-represented in a pathway, whereas others are randomly distributed to a metabolic network. This allows rapid assessment of potential alterations in a phenotype of interest in a hypothesis-generating study using untargeted metabolomics data [18].

Meta-analysis for pathway enrichment analysis or pathway-level meta-analysis is a powerful approach for capturing the biological signatures of a particular phenotype of interest across studies with heterogeneous settings [19]. Pathway-level meta-analysis of metabolomics data using GSEA has recently become feasible [20]. Using a computational method to predict functional activities from metabolic features and a pathway-level enrichment meta-analysis using GSEA may provide insight into the metabolic biosignature of phenotypes of interest.

Problematic reproducibility and minimal overlap of metabolic features across studies have hampered the investigation of the metabolic alterations in TB. There is an urgent need to develop a strategy to reliably capture the global metabolic biosignature of TB. Herein, we conducted a pathway-level GSEA-based meta-analysis of two pulmonary TB untargeted metabolomics data sets from South Korea and China. The analysis is a proof-of-concept of the ability of metabolomics meta-analysis using metabolic features to identify metabolic alterations in pulmonary TB.

## Results

### Data exploration reveals considerable metabolic feature changes in TB patients

**Fig 1:**
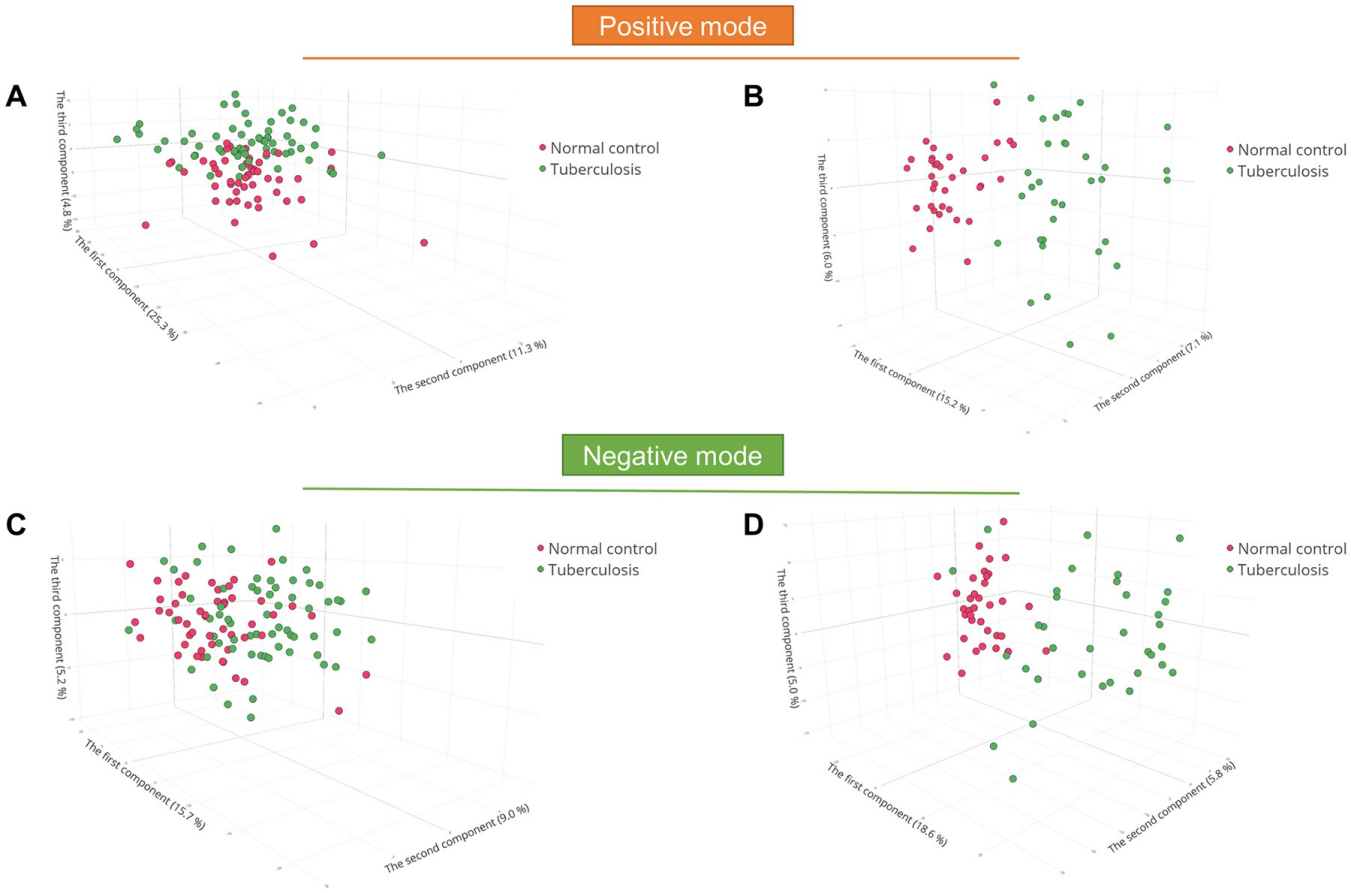
Data exploration and visualization. Principal component analysis of (A) cPMTb positive ion mode, (B) ST001231 positive ion mode, (C) cPMTb negative ion mode, and (D) ST001231 negative ion mode. TB, tuberculosis; NC, normal control.

We performed principal component analysis to examine and visualize the untargeted metabolomics data of the two studies in positive and negative ion modes. The three-dimensional score plots of cPMTb (positive ion mode), ST001231 (positive ion mode), cPMTb (negative ion mode), and ST001231 (negative ion mode) suggested comparatively clear separation of TB patients from their counterparts (Fig 1A–1D). However, the TB patients and cPMTb controls (NC) had a higher level of metabolome similarity than in ST001231.

Unpaired t-tests were also conducted, and the features were visualized using volcano plots. At a significance level of 0.05, only 218 features were upregulated, while 142 were downregulated in the TB group of cPMTb (positive ion mode, S1A Fig in S1 File). By contrast, 549 upregulated features and 412 downregulated features were found in the TB group in ST001231 (positive ion mode, S1B Fig in S1 File). Notably, few features had a high fold change in cPMTb, whereas many features had a high fold change in ST001231. Similar patterns were observed in negative ion mode (S1C and S1D Fig in S1 File).

### TB patients have distinct metabolome profiles

**Fig 2:**
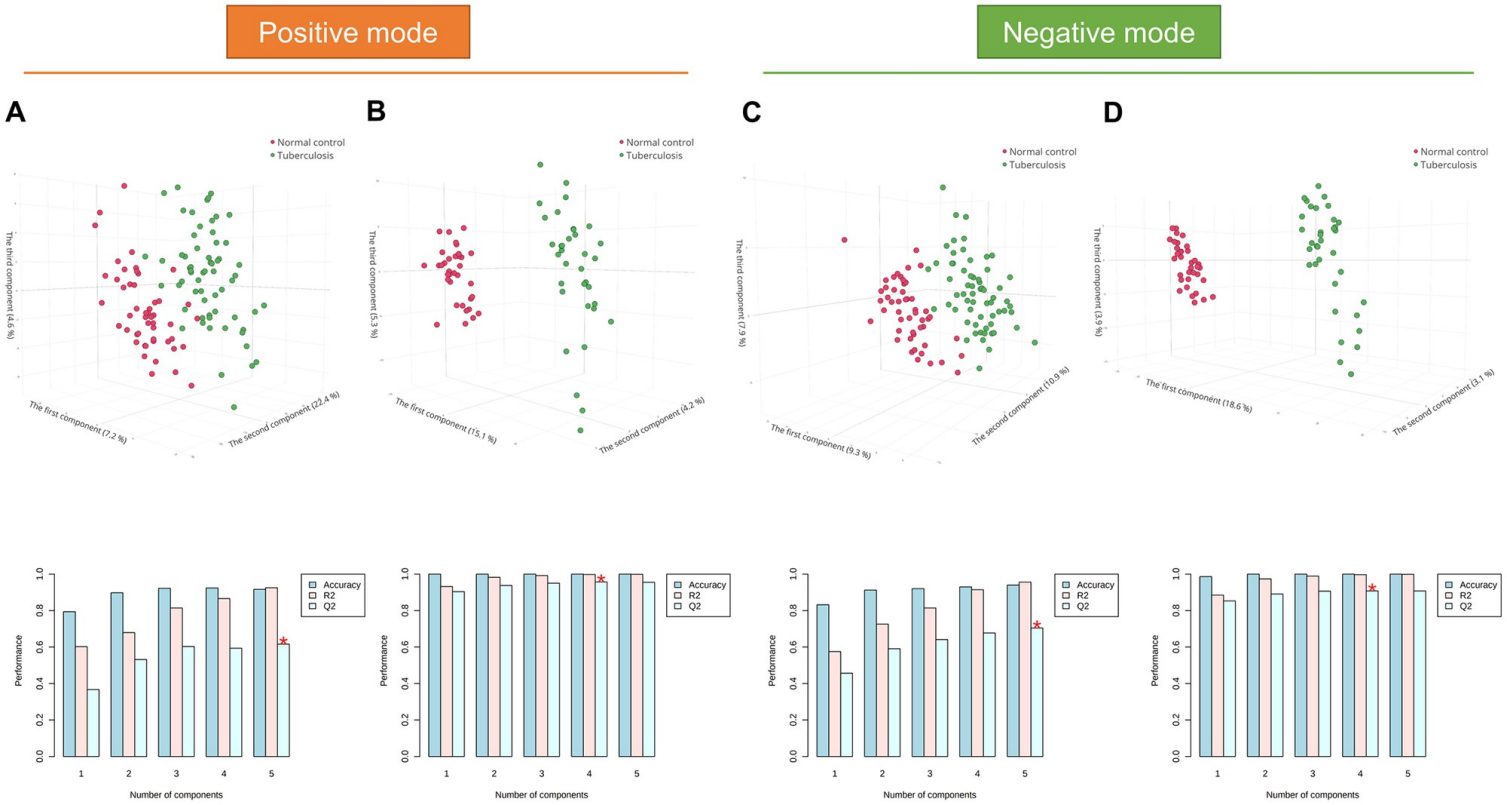
Partial least-squares discriminant analysis. (A) cPMTb positive ion mode. (B) ST001231 positive ion mode. (C) cPMTb negative ion mode. (D) ST001231 negative ion mode. * optimal value of Q^2^; TB, tuberculosis; NC, normal control.

Partial least-squares discriminant analysis and random forest analysis were used to examine whether the metabolic profiles could be used to classify TB patients and controls. In positive ion mode, the partial least-squares discriminant analysis models possessed excellent discriminatory performance. In particular, the optimal model in the cPMTb study contained five principal components with an accuracy, goodness-of-fit (R^2^), and goodness-of-prediction (Q^2^) of 0.90, 0.93, and 0.63, respectively (Fig 2A). Likewise, the optimal model in ST001231, which had four principal components, had an accuracy, R^2^, and Q^2^ of 1.00, 1.00, and 0.96, respectively (Fig 2B). Similar performance was observed in negative ion mode: cPMTb (accuracy, 0.94; R^2^, 0.96; Q^2^, 0.70) and ST001231 (accuracy, 1.00; R^2^, 1.00; Q^2^, 0.91) (Fig 2C and 2D). Remarkably, there were marked differences in metabolic profiles between TB patients and NCs in ST001231. The out-of-bag errors of the four random forest models were 0.12, 0.00, 0.13, and 0.00 for cPMTb (positive ion mode), ST001231 (positive ion mode), cPMTb (negative ion mode), and ST001231 (negative ion mode), respectively (S2 Fig in S1 File). These analyses collectively indicated that TB patients possess a distinct metabolome profile, compared with controls.

### Profound plasma metabolic alterations of pulmonary TB patients

**Fig 3:**
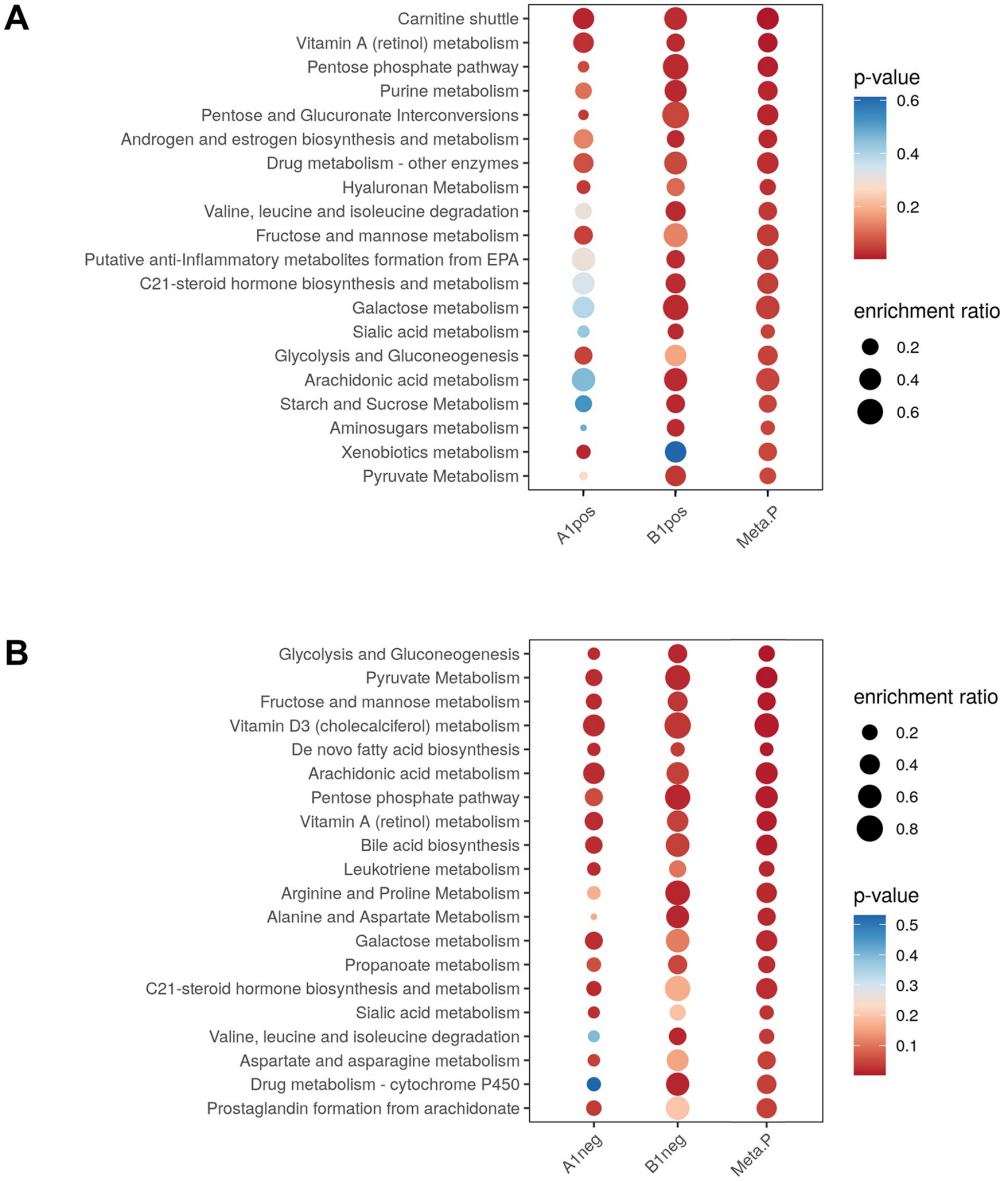
Pathway meta-analysis by gene set enrichment analysis. (A) Positive ion mode. (B) Negative ion mode. The enrichment factor of a pathway was calculated by dividing its number of significant hits by the expected number of hits.

Pathway-level meta-analysis was conducted separately in positive and negative ion modes. In positive ion mode, the meta-analysis revealed that 15 pathways had a combined *p*-value of < 0.05. They belonged to metabolic homeostasis, proinflammatory processes, and vitamin metabolism. The five pathways with the lowest combined *p*-values were “carnitine shuttle,” “vitamin A (retinol) metabolism,” “pentose phosphate pathway,” “purine metabolism,” and “pentose and glucuronate interconversions” (Fig 3A). Notably, only two pathways were significant in both individual studies among the significant pathways in the meta-analysis: “carnitine shuttle” and “vitamin A (retinol) metabolism.” Several pathways, including “pentose and glucuronate interconversions,” “hyaluronan metabolism,” and “fructose and mannose metabolism”—were enriched only in the cPMTb study (A1pos). By contrast, pathways, such as “sialic acid metabolism,” “purine metabolism,” and “androgen and estrogen biosynthesis and metabolism”—were significant only in ST001231 (B1pos). The heterogeneity of significant pathways among studies might be due to their relatively small sample sizes, sample heterogeneity, and use of different LC-MS platforms. More details are shown in S1 Table in S2 File.

Analysis of the data in negative ion mode yielded a greater number of significant pathways. Indeed, 24 pathways had a combined *p*-value of < 0.05 in the meta-analysis. Similar to the enriched pathways in positive ion mode, these belonged to proinflammatory processes, vitamin metabolism, metabolic homeostasis, amino acid-related metabolism, and some potentially novel pathways. Five of the pathways with the lowest combined *p*-values were “glycolysis and gluconeogenesis,” “pyruvate metabolism,” “fructose and mannose metabolism,” “vitamin D3 (cholecalciferol) metabolism,” and “de novo fatty acid biosynthesis” (Fig 3B). Among the significant pathways in the meta-analysis, eight were significantly enriched in both studies—the above-mentioned five pathways and “bile acid biosynthesis,” “arachidonic acid metabolism,” and “vitamin A (retinol) metabolism.” Seven pathways were significantly enriched only in cPMTb (A1neg), including “leukotriene metabolism,” “galactose metabolism,” “C21-steroid hormone biosynthesis and metabolism,” and “sialic acid metabolism.” In contrast, eight pathways—including “propanoate metabolism,” several amino acid-related pathways and “vitamin B3 (nicotinate and nicotinamide) metabolism”—were enriched only in ST001231 (B1neg). More details are shown in S2 Table in S2 File.

## Discussion

Meta-analysis enhances statistical power, reliability, and generalizability, especially in high-throughput data settings [21]. A feature-level meta-analysis provides more comprehensive information than secondary pooled analyses of a limited number of identified metabolites. Moreover, as mentioned above, metabolite identification remains a fundamental issue in metabolomics [22]. An analysis that forgoes metabolite identification significantly reduces the time (i.e., from days to hours) required to obtain valuable insights and derive actionable targets for the phenotype of interest. Therefore, we could focus more on the validation of potential biomarkers and the performance of experiments to delineate molecular mechanisms of disease.

In this study, a pathway-level GSEA-based meta-analysis of two pulmonary TB untargeted metabolomics data sets was conducted. The two included data sets had a significant degree of heterogeneity in clinical characteristics, which might affect the number of enriched pathways. Nevertheless, the meta-analysis provided considerable insight into global metabolic alterations in plasma from pulmonary TB patients. The results are pathophysiologically comparable with previous findings using conventional targeted methods in addition to novel metabolic alterations. The analysis is capable of suggesting biological processes that may be significantly influenced by the clinical characteristics of a cohort. Furthermore, the findings suggested that functional interpretation of metabolomics data at the pathway level can provide insights into the molecular signatures of TB patients. Importantly, biological speculations at the level of individual metabolites exhibit human-centric bias [23]. Below we discuss some of the most important findings.

“Vitamin A (retinol) metabolism” and “vitamin D3 (cholecalciferol) metabolism” were altered in the meta-analysis. Vitamin A deficiency is reportedly associated with an increased risk of incident TB among household contacts [24]. Vitamin A supplementation may boost immunity against TB [25], and vitamin A and zinc co-supplementation may improve outcomes [26]. Vitamin D3 deficiency is a risk factor for TB. Vitamin D3 supplementation may be associated with immune activation, and thus should improve treatment outcomes; however, this requires validation [26]. In addition, the “bile acid biosynthesis” and “purine metabolism” pathways were significantly altered in our TB patients compared to controls. These pathways may also be involved in host defense. Indeed, some bile acids inhibit the *in vitro* growth of *M*. *tuberculosis* [27]. Bile acid derivatives are also potential anti-TB agents [28], and purine metabolism in *M*. *tuberculosis* is a target for drug development [29, 30]. Furthermore, together with lipid metabolism, these pathways are reportedly linked to anti-TB drug-induced hepatotoxicity [31].

Notably, we observed significant systemic changes in the host (i.e., disease phenotype) due to TB infection. Proinflammatory lipid mediators and pro-resolving lipid mediators are associated with TB and strongly associated with TB comorbid type 2 diabetes. The arachidonic acid-derived leukotriene and prostaglandin families were reported to be the most abundant proinflammatory lipid mediators [32]. Our pathway analysis revealed significant enrichment of “arachidonic acid metabolism,” “leukotriene metabolism,” and “prostaglandin formation from arachidonate.” We also found various processes related to nutrients and oxidative stress, including “pyruvate metabolism,” “fructose and mannose metabolism,” “glycolysis and gluconeogenesis,” “*de novo* fatty acid biosynthesis,” and the metabolism of several amino acids. These findings are concur with a previous report that metabolic processes are involved in adaptations and/or interactions of the host and microbe during infection [33]. Medium-chain fatty acids are involved in protective immunity against *M*. *tuberculosis* [34]. Additionally, alteration of “pyruvate metabolism” might be linked to the increased catabolism and/or energy consumption observed in TB patients [35]. “Fructose and mannose metabolism” and “glycolysis and gluconeogenesis” in *M*. *tuberculosis* are reportedly affected by nutrient starvation. In addition, they are linked to central carbon metabolism, which is essential for the maintenance of metabolic homeostasis in *M*. *tuberculosis* [36]. For example, mycobacteria in phagosomes took up exogenous pyruvate more efficiently than glucose and the pyruvate was used as a carbon source for intracellular growth [37].

We also found some potentially important pathways associated with TB. In a study of the innate immune responses to *M*. *tuberculosis* using macrophages, Blischak *et al*. found a subset of genes specifically involved in infection, including protein-coding genes related to the regulation of sialic acid synthesis [38]. We found that “sialic acid metabolism” was altered in plasma from TB patients, and Isa *et al*. [39] reported an altered level of sialic acid in urine. Further studies are warranted to explore the role of sialic acid metabolism and the associated glycoproteins in the immune response, to understand the susceptibility of TB and potential therapeutic targets. “C21-steroid hormone biosynthesis and metabolism” was significantly changed in TB patients, and may be associated with pathological processes (e.g., host defense against TB infection) [40]. Finally, the roles of other pathways showing alterations in TB patients, such as “Vitamin B3 (nicotinate and nicotinamide) metabolism,” “Propanoate metabolism,” and “androgen and estrogen biosynthesis and metabolism,” remain to be elucidated.

This study had some limitations. First, the analysis was conducted with only two untargeted metabolomics data sets. The lack of data might impede the identification of subtle TB-associated metabolic disturbances. Second, similar to a recent study [41], the pathway annotations require validation. Nonetheless, the analysis validated the available pathological and biological evidence, suggesting its reliability. Third, blood-derived metabolomics studies cannot directly elucidate in vivo growth mechanisms or the mode of action of anti-TB drugs [13]. Instead, they are more suitable for applications related to host systematic molecular alterations. Finally, post hoc metabolite identification and individual quantification are required to evaluate the associations of metabolites with clinical TB manifestations.

## Conclusions

We showed that pathway meta-analysis of several studies can overcome cross-study inconsistency by increasing the power and generalizability of the results. In addition, pathologically comparable and novel metabolic alterations in plasma from pulmonary TB patients were described. Subsequent studies are needed to leverage these findings to discover novel diagnostic biomarkers, metabolism-informed clinical care, and metabolism-informed therapeutic development.

## Materials and methods

### Institutional review board statement

This study was approved by the Institutional Review Board of Korea University Guro Hospital (2017GR0012). All investigations were conducted in accordance with the principles of the Declaration of Helsinki. Informed consent was obtained from all subjects involved in the cPMTb study. Patients provided written informed consent for analysis of their blood and clinical data.

### Korean tuberculosis cohort characteristics

The samples used in this study were part of a multi-center TB cohort entitled Center for Precision Medicine for Tuberculosis (cPMTb). The biospecimens and data used for this study were provided by the Biobank of Korea University Guro Hospital, a member of Korea Biobank. Individuals with human immunodeficiency virus infection, chronic renal disease, chronic liver diseases, chronic lung diseases, and malignant diseases were excluded from the analysis. Eventually, plasma samples from 63 clinically diagnosed pulmonary TB patients and 50 normal controls were collected.

In the TB group, the mean age (± standard deviation) was 55 (± 16) years and 27% of the patients were women. Forty-eight patients (76%) had positive sputum smears and 14 patients (22%) had a chest cavity image on x-ray. In the controls, the mean age (± standard deviation) was 60 (± 10) years, and 58% of the controls were women.

### Chinese tuberculosis cohort characteristics

We downloaded data from TB patients and NC (Metabolomics Workbench, study ID ST001231) for the pathway-level meta-analysis with the cPMTb cohort to elucidate the metabolic profiles of pulmonary TB. In brief, the study involved 70 plasma samples of pulmonary TB (35 samples) and NC (35 samples). In the TB group, the age ranged from 18 to 64 years and 49% of the patients were women; of the patients, 86% had positive sputum smears and 17% had a chest cavity image on x-ray. The age of the NC group ranged from 23 to 60 years, and 31% of the controls were women. The untargeted metabolomics study was carried out by ultra-high-performance liquid chromatography coupled with Q Exactive mass spectrometer in positive and negative ion modes. More details are provided in the original publication [4].

### Chemicals and reagents

High-performance liquid chromatography-grade water, methanol, and acetonitrile (ACN) were from J.T. Baker (Phillipsburg, NJ, USA). Analytical-grade formic acid and ammonium acetate and the internal standard (cholic acid-d5), were purchased from Toronto Research Chemicals (Toronto, Canada). Authentic chemicals for establishing the in-house database were purchased from Sigma-Aldrich (St. Louis, MO, USA).

### Sample preparation

Blood samples were collected routinely on the day of enrollment in the overnight-fasted and medication-free state before treatment. Plasma was prepared by centrifuging the whole blood for 10 min at 4,500 rpm and stored at -80°C until analysis.

The extraction of metabolites from plasma was conducted in accordance with our established protocol [42]. In brief, 50 *μ*L of plasma were mixed with 150 *μ*L of ACN containing 5 *μ*g/mL cholic acid-d5 in a microcentrifuge Eppendorf tube. The mixture was vigorously vortexed for 5 min and centrifuged for 10 min at 13,000 rpm at 4°C; the supernatant was collected. An equal amount of each sample was collected and mixed to create a pooled quality control (QC) sample. All extracts were stored at -20°C and subsequently analyzed using a high-performance liquid chromatography quadrupole time of flight mass spectrometer.

### Instrumental conditions for untargeted metabolomics

The analysis was conducted as described previously with an Agilent 1200 series high-performance liquid chromatography (Agilent Technologies, Santa Clara, CA, USA) coupled to a 6530 Q-TOF mass spectrometer (Agilent Technologies) [42]. The autosampler was set at 4°C for all procedures. In positive ion mode, ACQUITY UPLC BEH C18 (100 × 2.1 mm, 1.7 *μ*m; Waters) was maintained at 40°C, and metabolite separation was conducted by binary gradient elution with a flow rate of 0.4 mL/min. Mobile phase A was water with 0.1% formic acid; mobile phase B was ACN with 0.1% formic acid. The gradient was 0 min, 2% B; 1 min, 2% B; 3 min, 20% B; 8 min, 90% B; 14 min, 90% B; 14.5 min, 2% B; 18 min, 2% B. Essential mass spectrometer parameters are given in S3 Table in S2 File. In negative ion mode, the ZIC-HILIC column (100 × 2.1 mm, 3.5 *μ*m; Merck, Darmstadt, Germany) was maintained at 35°C, and metabolite separation was conducted by binary gradient elution with a flow rate of 0.5 mL/min. Mobile phase A was ACN/water (5:95, v/v) with 10 mM ammonium acetate; mobile phase B was ACN/water (95:5, v/v) with 10 mM ammonium acetate. The gradient was 0 min, 99% B; 1 min, 99% B; 15 min, 50% B; 17 min, 50% B; 17.1 min, 99% B; and 22 min, 99% B. The mass spectrometer was operated using equivalent conditions to positive ion mode.

### Data preprocessing and alignment

The generated *.d raw files were converted to mzML files using ProteoWizard [43]. The mzML files were then submitted to MS-DIAL (version 4.60) [44] for peak detection, alignment, and annotation. Essential data processing parameters are given in S4 Table in S2 File. Features with sample average signals lower than fivefold above the blank average were removed. LOWESS signal correction across batches was applied to the aligned data set. Before subsequent statistical analyses, features with a relative standard deviation of ≥ 20% in QC samples were removed. Features with missing values in ≥ 50% of samples were also removed, otherwise imputed using feature-wise k-nearest neighbors. Finally, normalized and filtered features were log-transformed and Pareto scaled. Post-processing data treatment were conducted using MetaboAnalyst 5.0 [20].

The *.raw files from ST001231 were submitted directly to MS-DIAL (version 4.60) for peak detection, alignment, and annotation. Data processing parameters are given in S4 Table in S2 File. Because there were no blank samples, no feature removal based on blank information was applied. Features with missing values in ≥ 50% of the samples were removed, otherwise imputed using feature-wise k-nearest neighbors. Features with a relative standard deviation of ≥ 20% in QC samples were removed. Quantile normalization was employed for cross-sample normalization. Finally, the data were log-transformed and Pareto-scaled before subsequent analyses.

Normalized data are provided in S5-S8 Tables in S2 File.

### Data exploration and visualization

Principal component analysis was conducted to reduce data dimensionality, thus facilitating exploration and visualization of the data. The principal component analysis aims to find an orthogonal basis (or new axes) that can explain data variability and project observations onto a smaller subspace. In our study, *e*_1_, *e*_2_, *e*_3_ were the new axes (or eigenvectors corresponding to the three largest eigenvalues of the sample covariance matrix) and each observation $x\in R^{p}$ was converted to a vector (*x*^T^*e*_1_, *x*^T^*e*_2_, *x*^T^*e*_3_) and plotted in three-dimensional space.

### Statistical analysis

Multiple statistical methods were used to analyze the untargeted metabolomics data. For univariate analysis, unpaired t-tests were used. The adjusted *p*-value following the Benjamini-Hochberg procedure (i.e., a false discovery rate of 0.05) was used as the significance level. Partial least-squares discriminant analysis and random forest analysis (number of trees, 500; number of predictors, 50) were used to examine the class discrimination (i.e., TB and the counterpart) using metabolomics data. A 10-fold cross-validation procedure was used to measure classification performance.

### Pathway-level meta-analysis of metabolic features

The normalized and transformed data that contained *m/z* values, retention time (in seconds), and peak intensity for each ion mode were subjected to pathway-level meta-analysis. Before the pathway-level integration, the following calculations were performed: individual *m/z* statistics (i.e., t-test); putative metabolite annotation (mass tolerance, 10 ppm); and pathway prediction. Next, the *p*-values from individual studies were combined using Fisher’s method. Given individual *p*-values *p*_*i*_ from the *i*th hypothesis *i* = 1, …, *n*, the method aggregated them by:

$$X^{2}=-2\sum_{i=1}^{n}\text{log}\;p_{i},$$

which follows the chi-square distribution with degrees of freedom 2*n* under the null hypotheses.

GSEA was used for the pathway-level enrichment algorithm. In brief, the algorithm ranked all genes in data based on t-statistics and compared them to a prespecified gene set (or pathway), termed S. If top-ranked genes (i.e., large t-statistics) had many overlaps with S, such that the enrichment score increased, then S was regarded as an active pathway. The *Homo sapiens* (human) [MFN] (combined KEGG, BiGG, and Edinburgh) was used as the pathway library for analysis. A pathway with a combined *p*-value of < 0.05 was considered statistically significant.

## Supporting information

S1 File Volcano plots of metabolic features and the random forest classification of the two studies. (DOCX)

S2 File Supplementary method and materials and data. (XLSX)
