# Supplementary material for: Molecular perturbations in pulmonary tuberculosis patients identified by pathway-level analysis of plasma metabolic features
Source: PLoS One. 2022 Jan 24;17(1):e0262545. doi: 10.1371/journal.pone.0262545 (PMC8786114; doi:10.1371/journal.pone.0262545)

**Molecular Perturbations in Pulmonary Tuberculosis Patients Identified by Pathway-level Analysis of Plasma Metabolic Features**

Nguyen Phuoc Long^1,2^, Da Young Heo^2^, Seongoh Park^3^, Nguyen Thi Hai Yen^1,2^, Yong-Soon Cho^1,2^, Jae-Gook Shin^1,2,4^, Jee Youn Oh^5,*^, Dong-Hyun Kim^2,*^

^1^ Center for Personalized Precision Medicine of Tuberculosis, Inje University College of Medicine, Busan 614735, Republic of Korea

^2^ Department of Pharmacology and PharmacoGenomics Research Center, Inje University College of Medicine, Busan 614735, Republic of Korea

^3^ Department of Statistics, Sungshin Women’s University, Seoul 02844, Republic of Korea

^4^ Department of Clinical Pharmacology, Inje University Busan Paik Hospital, Busan 614735, Republic of Korea

^5^ Division of Pulmonary, Allergy and Critical Care Medicine, Department of Internal Medicine, Korea University Guro Hospital, Seoul, Republic of Korea

*Corresponding author

E-mail: [dhkim@inje.ac.kr](mailto:dhkim@inje.ac.kr) (DHK) and [happymaria0101@hanmail.net](mailto:happymaria0101@hanmail.net) (JYO)

**Fig S1.** **Volcano plots of metabolic features of the two studies.** Volcano plots of (A) cPMTb positive ion mode, (B) ST001231 positive ion mode, and (C) cPMTb negative ion mode. (D) ST001231 negative ion mode.


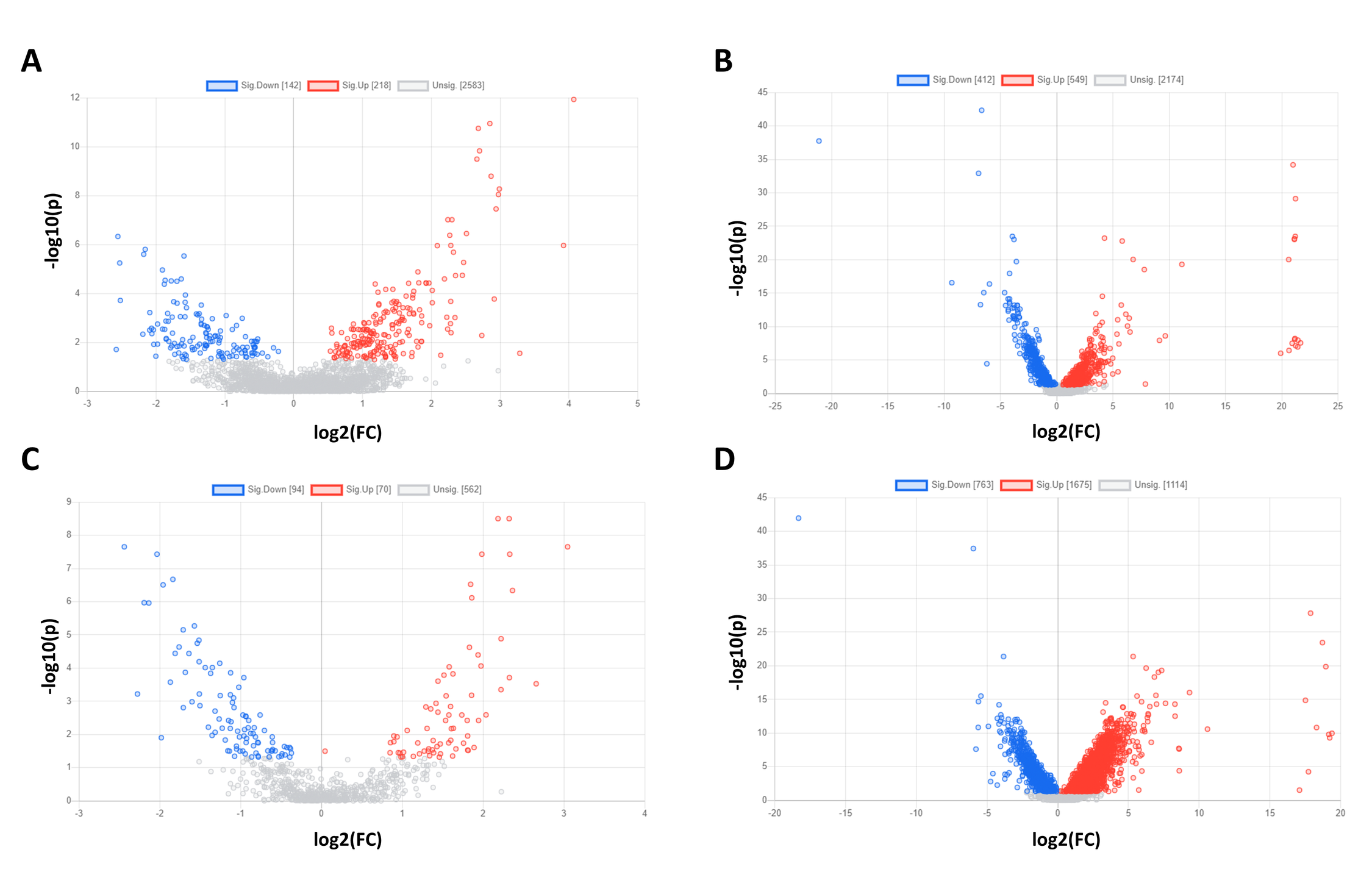


**Fig S2. The Random Forest Classification of the two studies.** (A) cPMTb positive ion mode. (B) ST001231 positive ion mode. (C) cPMTb negative ion mode. (D) ST001231 negative ion mode. TB, tuberculosis; NC, normal control.


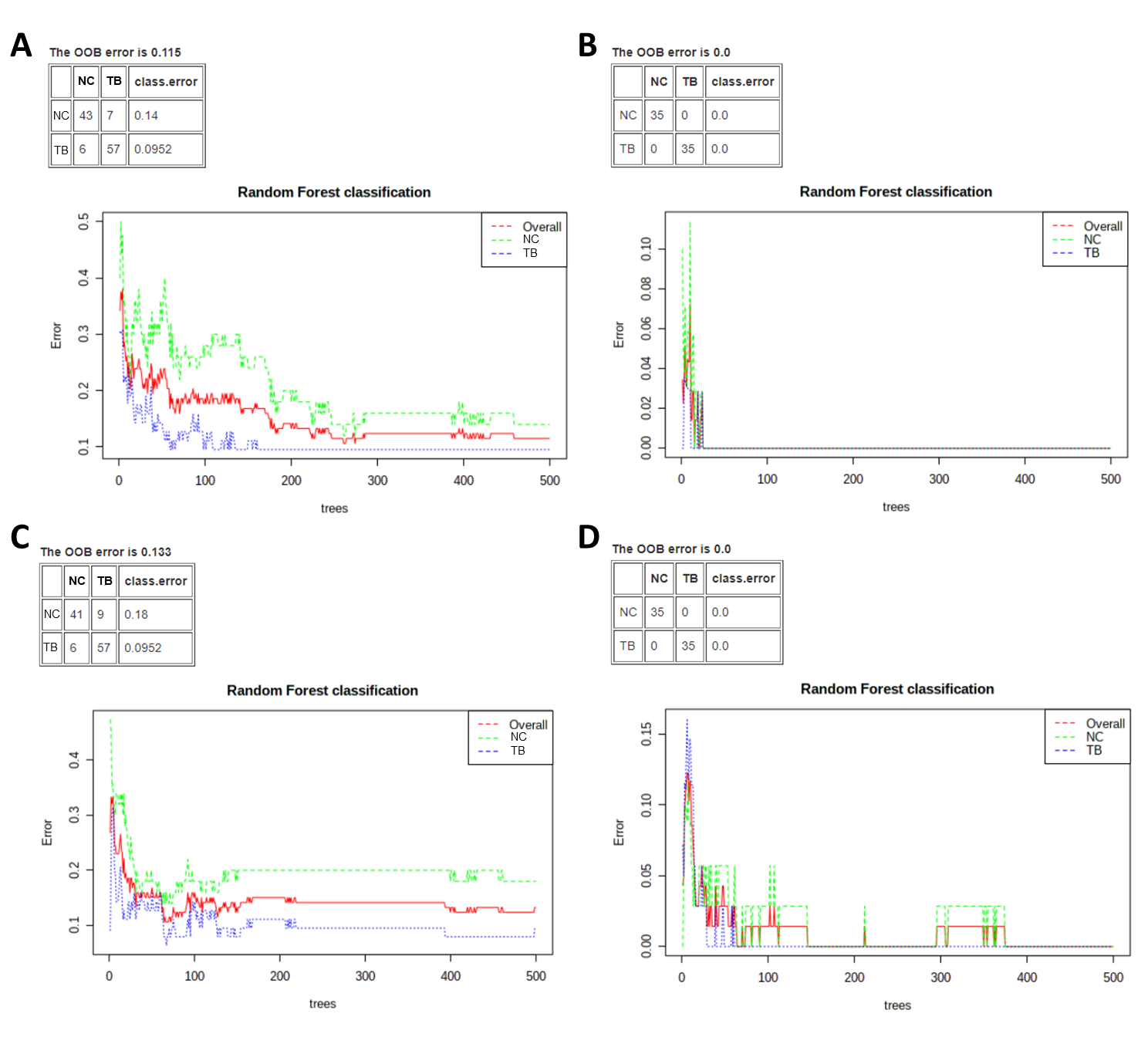

Supplement: S1 File — (DOCX) [file pone.0262545.s001.docx]
